# Supplementary material for: Characterization of the Radiation Desiccation Response Regulon of the Radioresistant Bacterium Deinococcus radiodurans by Integrative Genomic Analyses
Source: Cells. 2021 Sep 25;10(10):2536. doi: 10.3390/cells10102536 (PMC8533742; doi:10.3390/cells10102536)
Supplement: Supplementary file 1 [file cells-10-02536-s001.zip › cells-1325232-supplementary publish check/upload/Figure S1-S6.pdf]

Supporting Figures

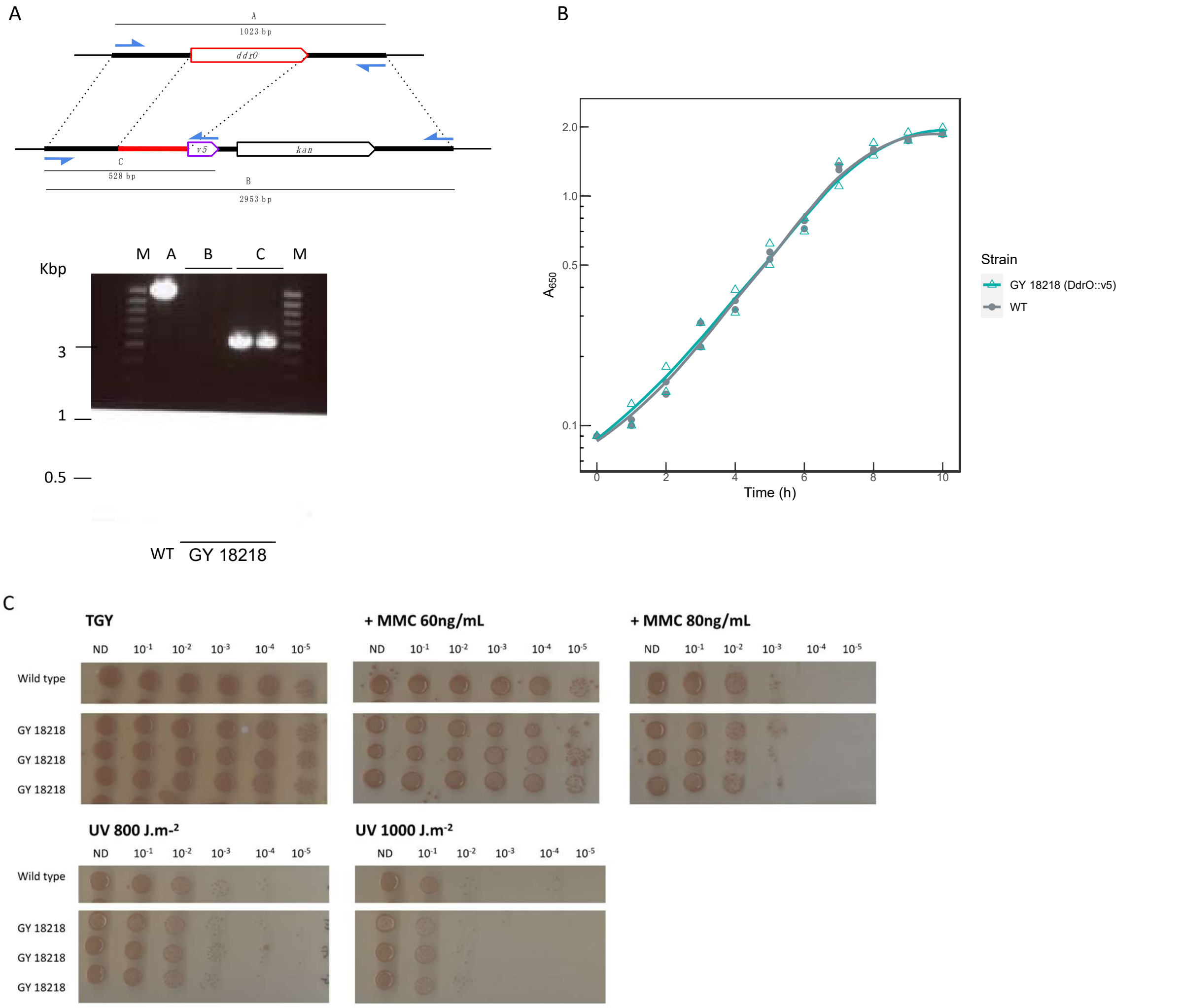

Figure S1: A *ddrO* allele replacement by a gene expressing the recombinant DdrO-V5 protein did not modify the growth rate.

(A) Schematic representation and test of the homozygosity of the *ddrO*::V5Ω*kan* strain (GY 18218 strain). Diagnostic PCRs for the replacement of *ddrO* wild-type allele by *ddrO::v5Ωkan* are designated by blue arrows.

(B) Growth curves of the *ddrO-V5::Ωkan* and wild type strains.

(C**)** Cells expressing DdrO-V5 are as resistant to MMC and UV as is the wild type strain. Wild type (R1), and 3 independent clones of GY 18218 strain expressing the recombinant DdrO-V5 grown to an A_{650nm} = 0.3 were serially diluted in TGY2X broth, and aliquots (10 µl) of each dilution were spotted on TGY agar plates or plates containing 60 or 80 ng/ mL MMC, or were exposed to UV radiation at the indicated UV doses before incubation at 30°C for 3±5 days.

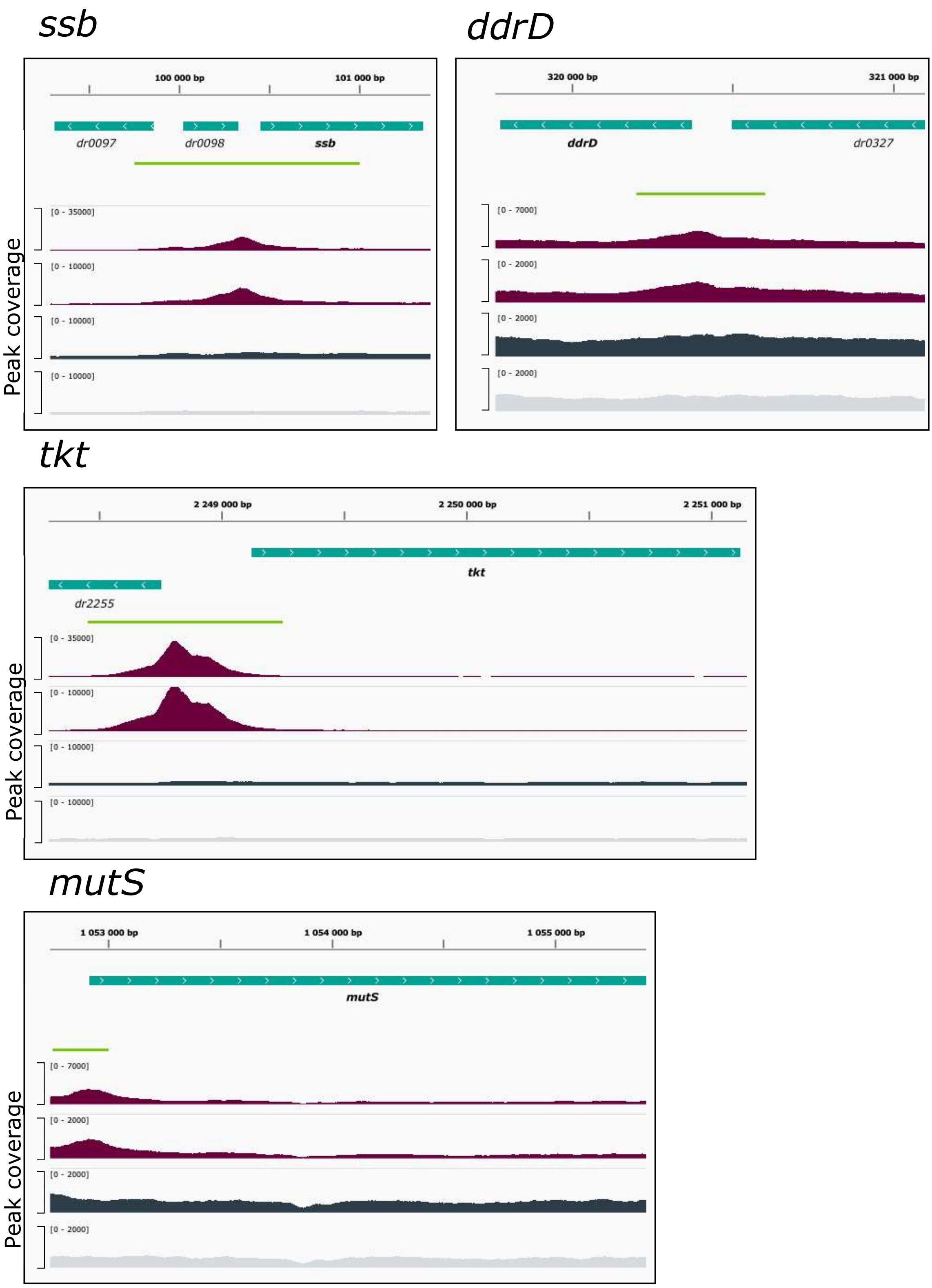
ssb
100 000 bp
101 000 bp
dr0097
dr0098
ssb
[0 - 35000]
[0 - 10000]
[0 - 10000]
[0 - 10000]
Peak coverage
ddrD
320 000 bp
321 000 bp
ddrD
dr0327
[0 - 7000]
[0 - 2000]
[0 - 2000]
[0 - 2000]
tkt
2 249 000 bp
2 250 000 bp
2 251 000 bp
tkt
dr2255
[0 - 35000]
[0 - 10000]
[0 - 10000]
[0 - 10000]
Peak coverage
mutS
1 053 000 bp
1 054 000 bp
1 055 000 bp
mutS
[0 - 7000]
[0 - 2000]
[0 - 2000]
[0 - 2000]
Peak coverage

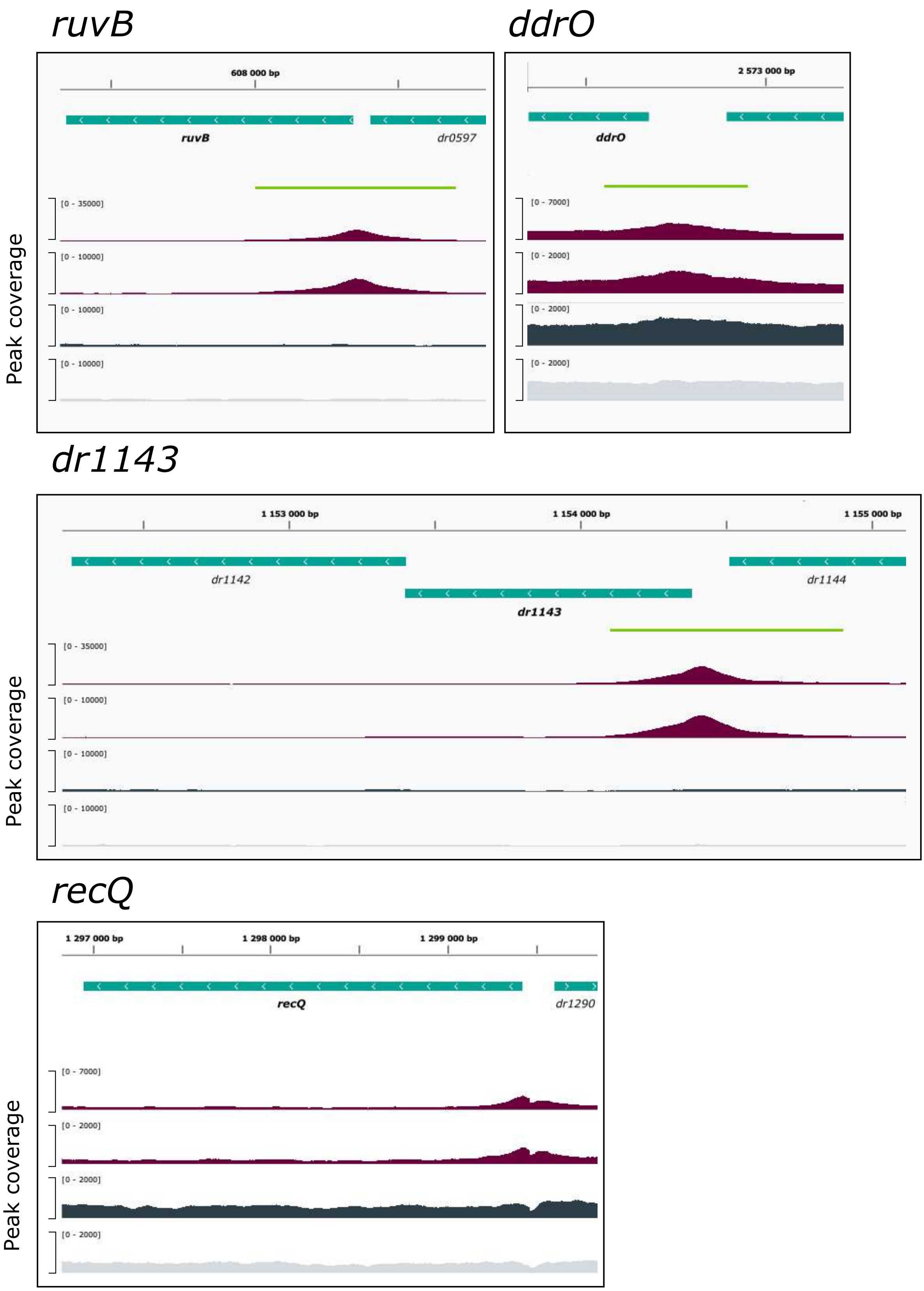

ruvB
608 000 bp
ruvB
dr0597
[0 - 35000]
[0 - 10000]
[0 - 10000]
[0 - 10000]
Peak coverage
ddrO
2 573 000 bp
ddrO
[0 - 7000]
[0 - 2000]
[0 - 2000]
[0 - 2000]
dr1143
1 153 000 bp
1 154 000 bp
1 155 000 bp
dr1142
dr1144
dr1143
[0 - 35000]
[0 - 10000]
[0 - 10000]
[0 - 10000]
Peak coverage
recQ
1 297 000 bp
1 298 000 bp
1 299 000 bp
recQ
dr1290
[0 - 7000]
[0 - 2000]
[0 - 2000]
[0 - 2000]
Peak coverage

ddrF

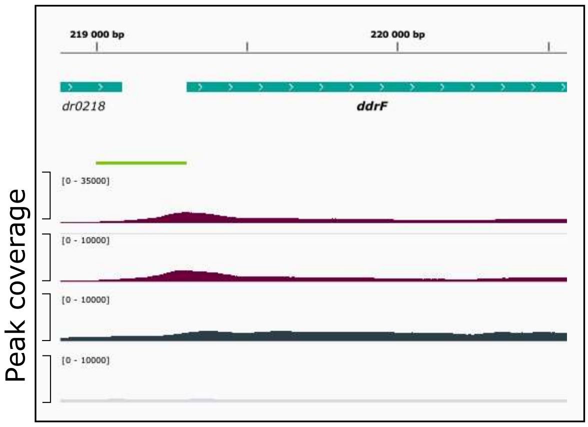

uvrB

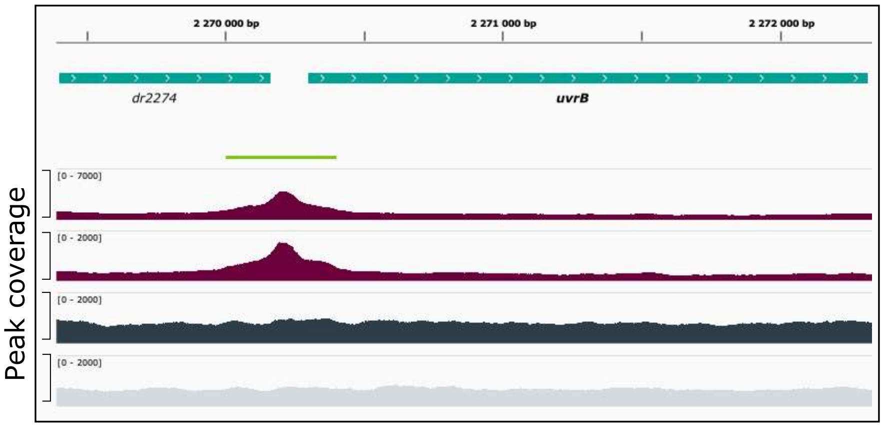

cinA – ligT - recA

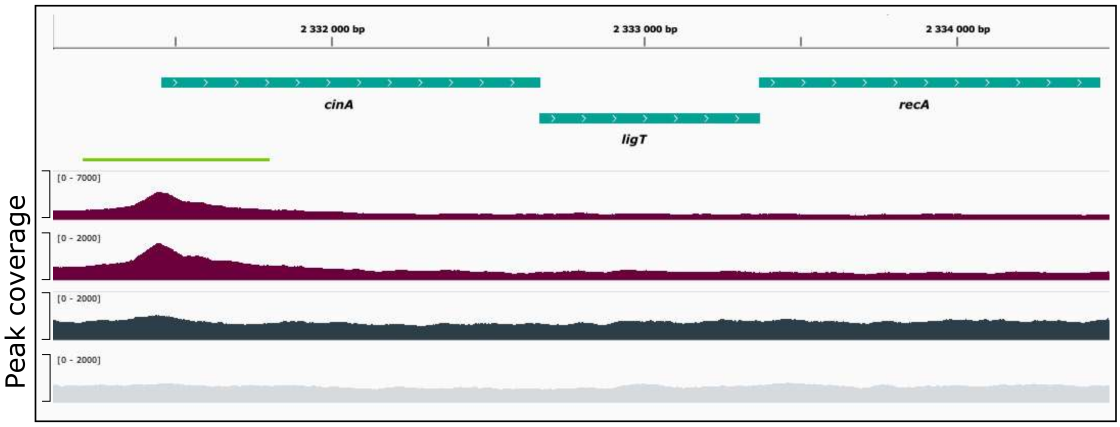

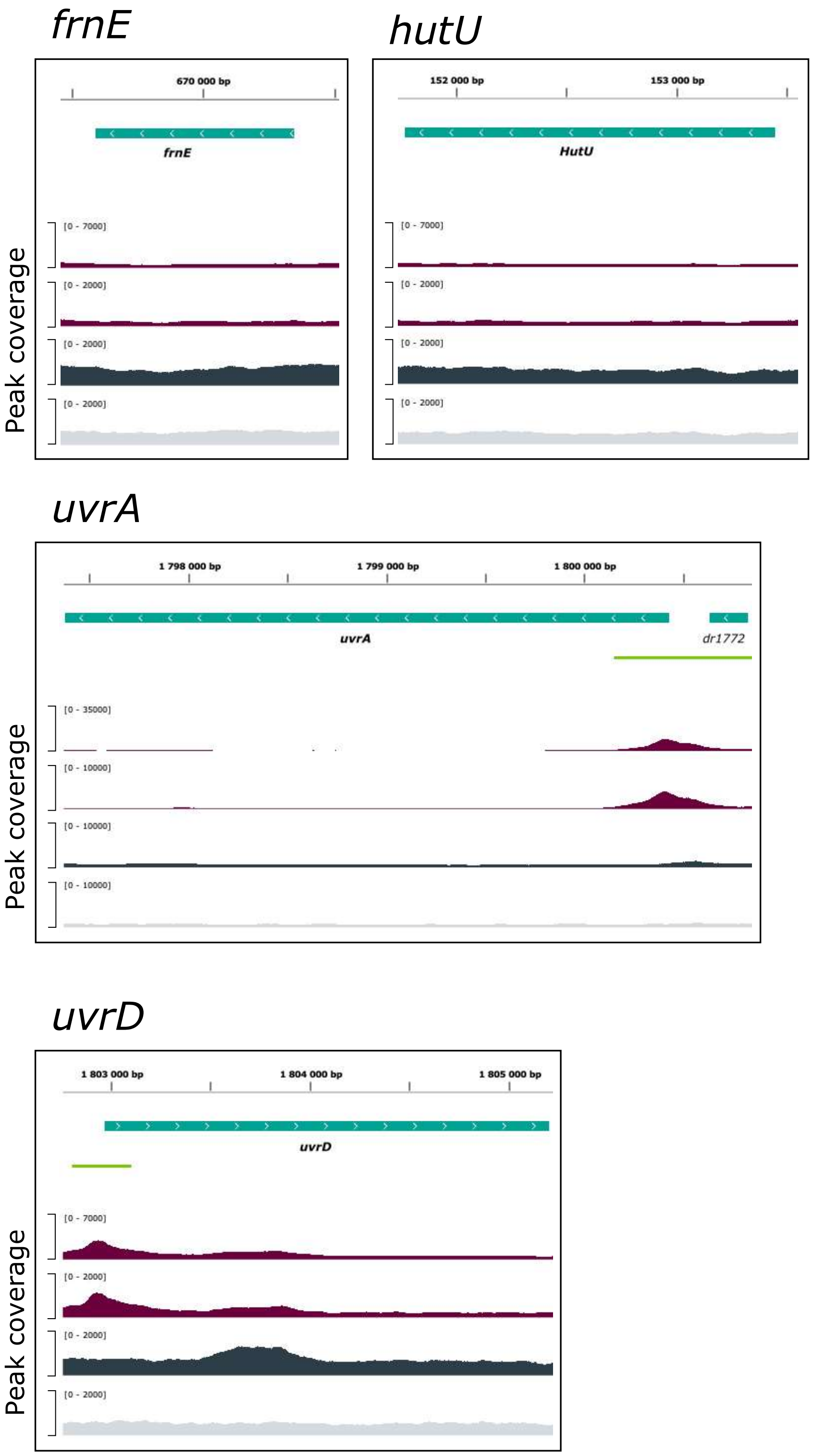
frnE
670 000 bp
frnE
[0 - 7000]
[0 - 2000]
[0 - 2000]
[0 - 2000]
Peak coverage
hutU
152 000 bp
153 000 bp
HutU
[0 - 7000]
[0 - 2000]
[0 - 2000]
[0 - 2000]
uvrA
1 798 000 bp
1 799 000 bp
1 800 000 bp
uvrA
dr1772
[0 - 35000]
[0 - 10000]
[0 - 10000]
[0 - 10000]
Peak coverage
uvrD
1 803 000 bp
1 804 000 bp
1 805 000 bp
uvrD
[0 - 7000]
[0 - 2000]
[0 - 2000]
[0 - 2000]
Peak coverage

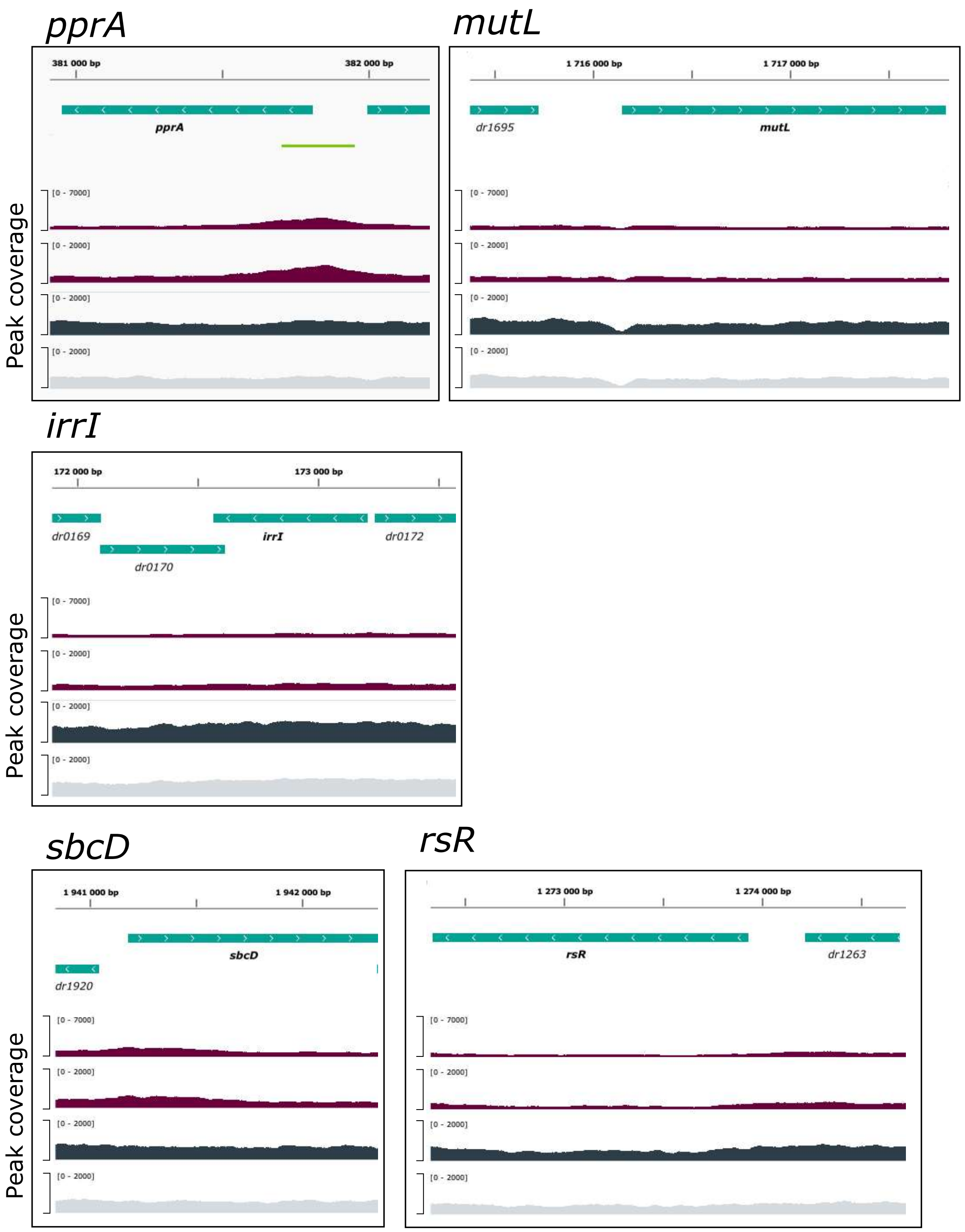

Figure S2. Visualization, through IGV, of the tag density profiles for 20 genes reported as belonging to the RDR regulon. Tag density profiles are illustrated for 2 IP (purple), the Input (dark grey) and the mock (light grey). The green lines indicated the size of each peak identified by bPeaks. Genes are represented by green boxes, their location on either strand is indicated by > (strand +) and < (strand -).

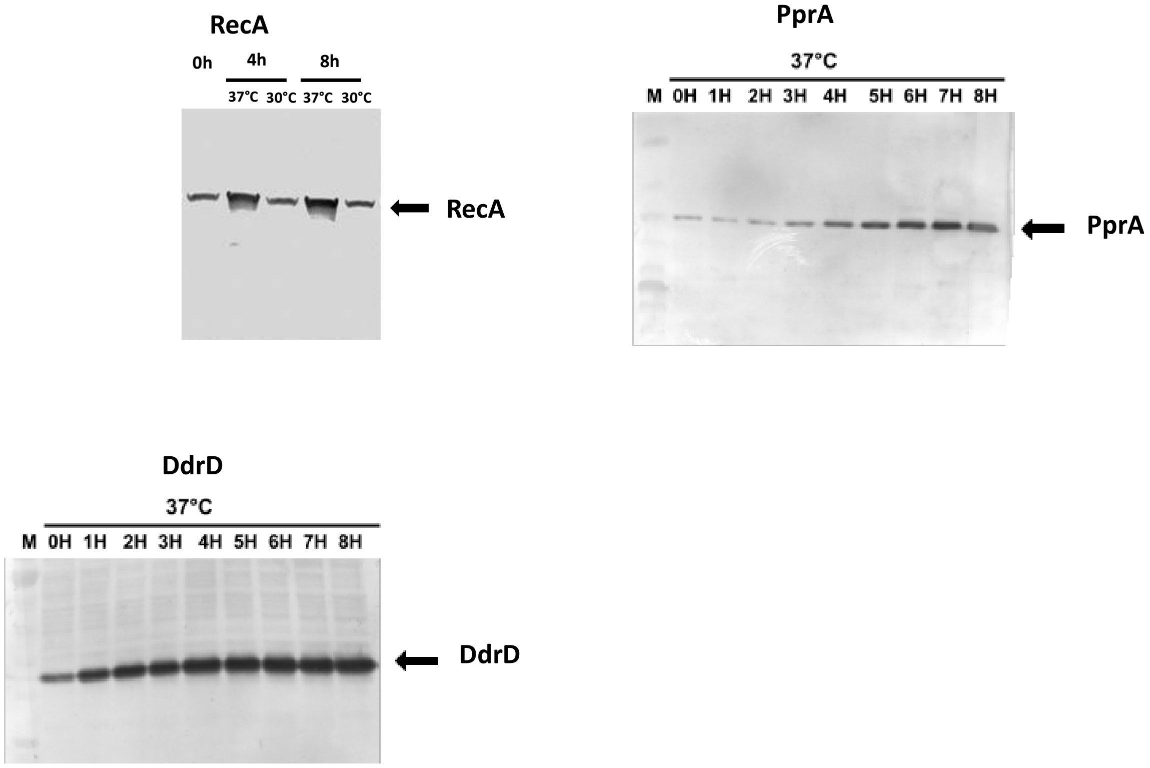

Figure S3. Western-blot analysis of the expression of the recombinant RecA-HA, PprA-HA and DdrD-HA proteins in D37 at 37°C.

At each time point, aliquots of cells were taken and cell extracts were subjected to SDS-PAGE and analyzed by Western blotting with anti-HA antibodies. For DdrD-HA, RecA-HA and PprA-HA 10 µg, 10 µg and 5 µg of proteins, respectively, were loaded in each well.

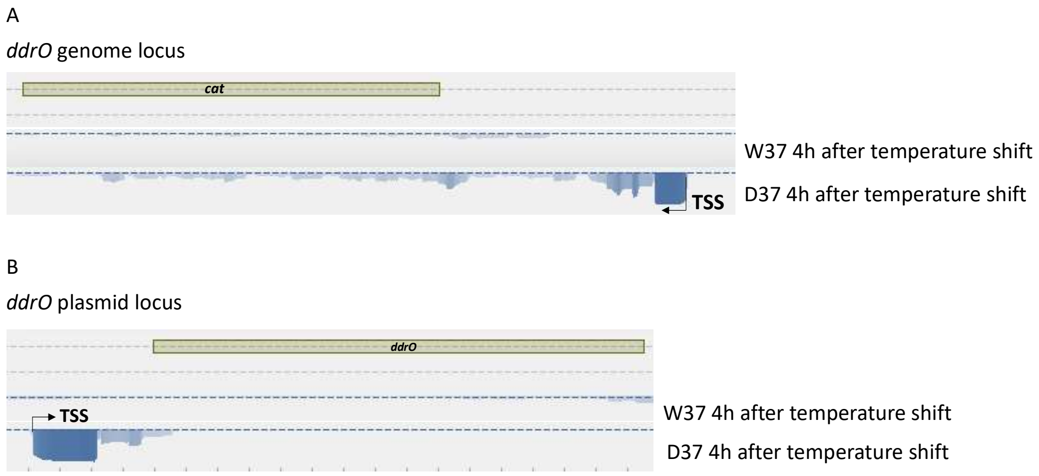

Figure S4. Visualization using Tablet software [73] of the DdrO gene reads mapping at 4 h at the *D. radiodurans ddrO* genome locus and on the replication plasmids in strains W37 and D37, respectively.

The position of the *ddrO* gene on the plasmids as well as the chloramphenicol resistance cassette *(cat)* used for genomic *ddrO* allele replacement are shown by brown boxes. The blue profiles show the densities of reads mapping on the 5′UTR at each locus. Only a few sequences mapped with the *ddrO* CDS in W37, since DdrO represses the expression of its own gene. After 4 h at 37°C, reads mapped with the 5′UTR of the *ddrO* gene in D37 as well as with the same locus located in the plasmid because of the expression of this duplicated DNA segment, but a very few sequences were observed mapping inside the CDS since D37 have lost the thermosensitive $repU_{Ts}$ plasmid.

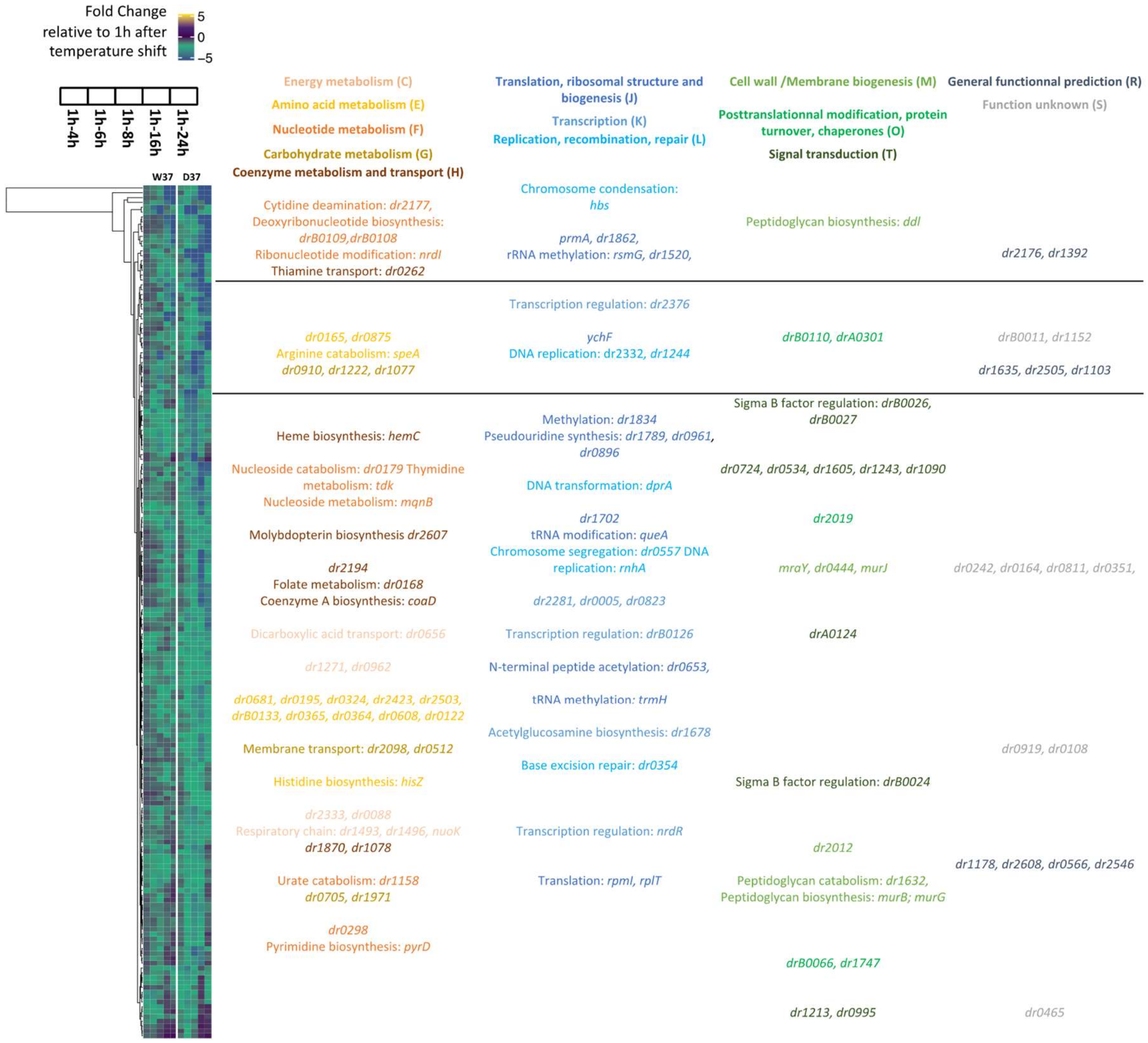

Figure S5. Hierarchical clustering of genes whose expression is specifically downregulated in response to DdrO depletion.

The 176 genes differentially expressed between times 1 h-6 h and 1 h-16 h in W37 strain in less than or in two comparisons (DE ≤ 2) and in more than three comparisons in the D37 strain (DE > 3) were hierarchically clustered according to their temporal expression. Only several genes representing some COG categories are shown.

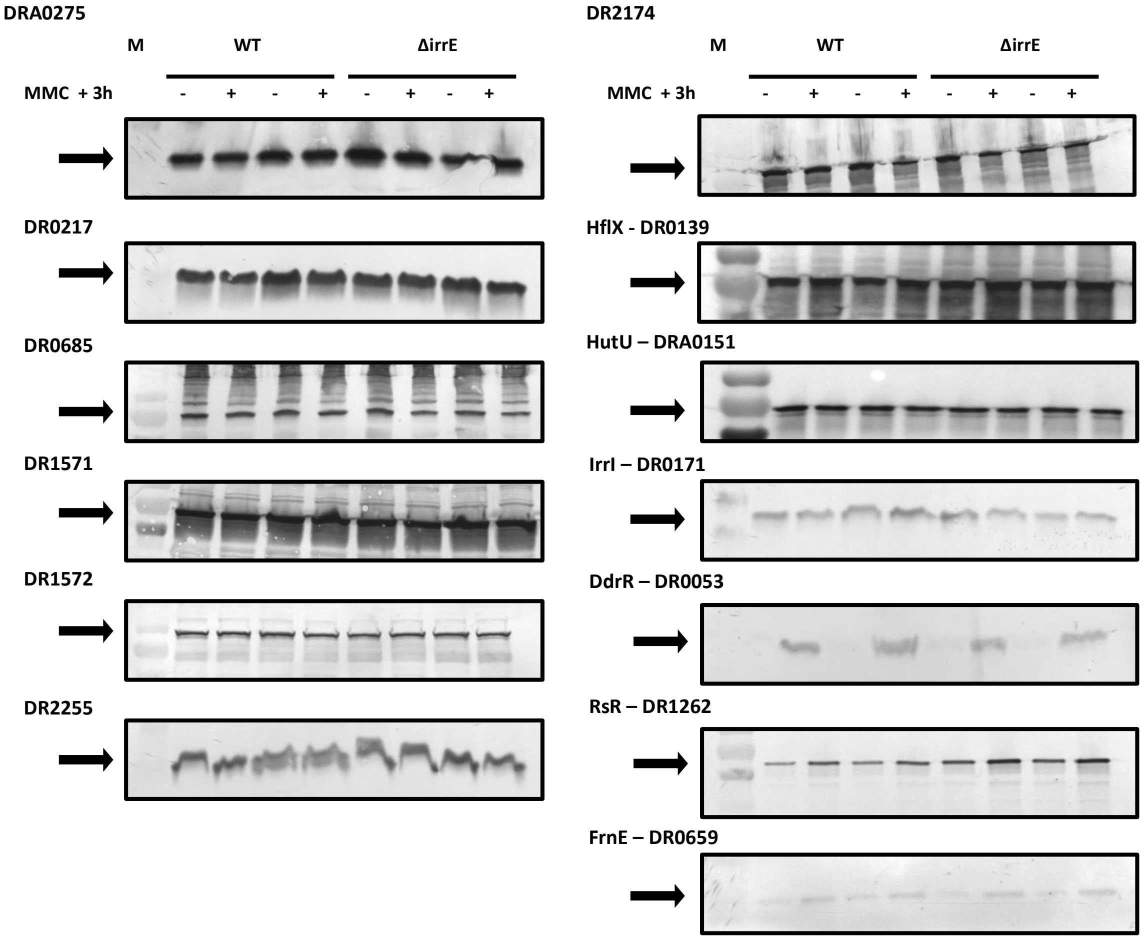

Figure S6. Expression of several proteins after exposure to MMC in an IrrE and DdrO independent manner. WT or *ΔirrE* cells expressing recombinant V5 or HA protein were incubated (+) or not (-) with MMC (1 µg/ ml) at 30 °C for 1 h. The expression of several previously described DdrO targets genes, such as HutU, IrrI, DdrR, RsR and FrnE were also analyzed after exposure to MMC. Cell extracts were subjected to SDS-PAGE and analyzed by Western blotting with anti-V5 or anti-HA antibodies. Fifteen µg of proteins were loaded in each well, except for DRA0275 and DR0217 (5 µg).
