# Supplementary material for: Characterization of the Radiation Desiccation Response Regulon of the Radioresistant Bacterium Deinococcus radiodurans by Integrative Genomic Analyses
Source: Cells. 2021 Sep 25;10(10):2536. doi: 10.3390/cells10102536 (PMC8533742; doi:10.3390/cells10102536)
Supplement: Supplementary file 1 [file cells-10-02536-s001.zip › cells-1325232-supplementary publish check/upload/File S1.pdf]

S1 File

```
###
# Gaëlle Lelandais <gaelle.lelandais@universite-paris-saclay.fr>
# Fabrice Confalonieri <fabrice.confalonieri@universite-paris-saclay.fr>
###
###### ----------------------------------------------------------------------
# NOTE from the authors :
# This script provides detailed information about the process which was applied
# to obtain the results presented in the main text and supplementary data files.
# Note that it does not allow the direct replication of all calculations,
# since some sections required executions of external scripts. Since, it
# has the interest to give a general overview of the different steps which were
# done to mine our dataset and hence define without a priori different lists of
# candidate genes to be DDr0 targets.
###### ----------------------------------------------------------------------

#####
# Part 1 : Mining ChIP-seq results (peak calling with bPeaks)
#####

if(0){

# Get bPeaks library
library(bPeaks)

# Data reading - Input is used as control here
Rep1 = dataReading("./data/IP-GY-18220-1_S1_all.genomeCoverage.txt",
               "./data/Input-GY-18220-1_S4_all.genomeCoverage.txt")
Rep2 = dataReading("./data/IP-GY-18220-2_S2_all.genomeCoverage.txt",
               "./data/Input-GY-18220-1_S4_all.genomeCoverage.txt")
Rep3 = dataReading("./data/IP-GY-18220-3_S13_all.genomeCoverage.txt",
               "./data/Input-GY-18220-1_S4_all.genomeCoverage.txt")

# Data reading - Mock is used as control here
Rep1m = dataReading("./data/IP-GY-18220-1_S1_all.genomeCoverage.txt",
               "./data/Mock-IP-GY-9613_S5_all.genomeCoverage.txt")
Rep2m = dataReading("./data/IP-GY-18220-2_S2_all.genomeCoverage.txt",
               "./data/Mock-IP-GY-9613_S5_all.genomeCoverage.txt")
Rep3m = dataReading("./data/IP-GY-18220-3_S13_all.genomeCoverage.txt",
               "./data/Mock-IP-GY-9613_S5_all.genomeCoverage.txt")

# -- The set of parameters is (2, 2, 0.5, 0.9) for all bPeaks analyses
res1 = bPeaksAnalysis(Rep1$IPdata, Rep1$controlData,
                IPcoeff = 2, controlCoeff = 2, log2FC = 0.5, averageQuantiles = 0.9,
                resultName = "Rep1", peakDrawing = F, promSize = 800,
                withoutOverlap = FALSE)

res2 = bPeaksAnalysis(Rep2$IPdata, Rep2$controlData,
                IPcoeff = 2, controlCoeff = 2, log2FC = 0.5, averageQuantiles = 0.9,
                resultName = "Rep2", peakDrawing = F, promSize = 800,
                withoutOverlap = FALSE)

res3 = bPeaksAnalysis(Rep3$IPdata, Rep3$controlData,
                IPcoeff = 2, controlCoeff = 2, log2FC = 0.5, averageQuantiles = 0.9,
                resultName = "Rep3", peakDrawing = F, promSize = 800,
                withoutOverlap = FALSE)

res4 = bPeaksAnalysis(Rep1m$IPdata, Rep1m$controlData,
                IPcoeff = 2, controlCoeff = 2, log2FC = 0.5, averageQuantiles = 0.9,
                resultName = "Rep1m", peakDrawing = F, promSize = 800,
                withoutOverlap = FALSE)

res5 = bPeaksAnalysis(Rep2m$IPdata, Rep2m$controlData,
                IPcoeff = 2, controlCoeff = 2, log2FC = 0.5, averageQuantiles = 0.9,
                resultName = "Rep2m", peakDrawing = F, promSize = 800,
                withoutOverlap = FALSE)

res6 = bPeaksAnalysis(Rep3m$IPdata, Rep3m$controlData,
                IPcoeff = 2, controlCoeff = 2, log2FC = 0.5, averageQuantiles = 0.9,
                resultName = "Rep3m", peakDrawing = F, promSize = 800,
                withoutOverlap = FALSE)

# -- All results were moved in a separate folder (named "bPeaks")
```

```
#####
# Part 2 : Combine bPeaks results into a single list of peaks
#####

# NOTE : This is done with an external SHELL script :
# manage_peaks.sh
# --> The file FinalPeaks.bed is obtained.

# Get final list of peaks and rename the peaks to simplify following
# analyses
peakList = read.table("FinalPeaks.bed", sep = "\t")
peakList = cbind(peakList, paste0("FinalPeaks_", 1:nrow(peakList)))
row.names(peakList) = peakList[,4]

write.table(peakList, file = "FinalPeaks_names.bed",
          sep = "\t", quote = F, row.names = F, col.names = F)

# Locate the peaks according to CDS positions
# -- CDS information
CDSinfo = as.matrix(read.table("./data/ConsA_III_CDS_bPeaks.txt",
                              sep = "\t", header = T))

# Get a specific function to search for peaks located near ATG position.
# This function is derived from the bPeaks package, it allows to search
# peak in a location from -500 to +100 (refered to ATG).
source("peakLocationNOVOREP.R")
peakLocationNOVOREP(bedFile = "FinalPeaks_names.bed", cdsPositions = CDSinfo,
                   outputName = "FinalPeaks_location",
                   promSize1 = 500, promSize2 = 100)

# Results are in file "FinalPeaks_location_peakLocation_inPromoters"
# --> 116 peaks are found.

# List of peaks in promoters
promPeaks = unique(read.table("FinalPeaks_location_peakLocation_inPromoters.txt")[,1])

# Get genomic location of promPeaks
peakList2 = peakList[promPeaks,]
write.table(peakList2, file = "FinalPeaks_promoters.bed",
          sep = "\t", quote = F, row.names = F, col.names = F)

# Get genomic sequences of peaks (in promoters)
source("Script_extractSeq.R")

# -- All results were moved in the folder names "ChIP_results".

}

#####
# Part 3 : Combine ChIP-seq results with transcriptomics data
#####

# Transcriptomic data
expData = as.matrix(read.csv("data_transcriptome2.txt",
                  sep = "\t", row.names = 1, header = T))

# Convert ratio into logFC
expData2 = expData
expData2[,seq(1,ncol(expData2), by = 2)] = log2(expData[,seq(1,ncol(expData), by = 2)])

# Add peak names in the table (and peak location)
promPeaks = read.table("FinalPeaks_location_peakLocation_inPromoters.txt", sep = "\t")

numPeaks    = NULL
whichPeaks  = NULL

# To store peak coordinates
peakCoord = as.matrix(read.table("FinalPeaks_promoters.bed", row.names = 4))
coordPeaks  = NULL

for(i in 1:nrow(expData2)){
  if(sum(promPeaks[,3] == row.names(expData2)[i]) == 0){
    numPeaks   = c(numPeaks, 0)
    whichPeaks = c(whichPeaks, NA)
    coordPeaks = c(coordPeaks, NA)
  }else if(sum(promPeaks[,3] == row.names(expData2)[i]) == 1){
```

```
    numPeaks   = c(numPeaks, 1)
    whichPeaks = c(whichPeaks, promPeaks[promPeaks[,3] == row.names(expData2)[i],1])
    coordPeaks = c(coordPeaks, paste(peakCoord[promPeaks[promPeaks[,3] == row.names(expData2)[i],1],], collapse = "|"))
  }else if(sum(promPeaks[,3] == row.names(expData2)[i]) > 1){
    numPeaks   = c(numPeaks, sum(promPeaks[,3] == row.names(expData2)[i]))
    whichPeaks = c(whichPeaks, paste(promPeaks[promPeaks[,3] == row.names(expData2)[i],1], collapse = "|"))
    # WARNING !! In case of multiple peaks, coordinates are note stored
    coordPeaks = c(coordPeaks, paste(peakCoord[promPeaks[promPeaks[,3] == row.names(expData2)[i],1],], collapse = "|"))
  }

# end of for()
}

# Differential expression ?
diffRes  = as.matrix(expData2[,11:20])
diffResC = expData2[,1:10]

# Threshold values
T1 = 1 # logFC
T2 = 0.01 # p-value

# Create tables to keep DE information
resTableUP    = matrix(NA, nrow = nrow(diffRes), ncol = (ncol(diffRes) / 2))
resTableDOWN  = matrix(NA, nrow = nrow(diffRes), ncol = (ncol(diffRes) / 2))

resTableUPc    = matrix(NA, nrow = nrow(diffRes), ncol = (ncol(diffRes) / 2))
resTableDOWNc  = matrix(NA, nrow = nrow(diffRes), ncol = (ncol(diffRes) / 2))

row.names(resTableUP)   = row.names(diffRes)
row.names(resTableDOWN) = row.names(diffRes)
row.names(resTableUPc)   = row.names(diffRes)
row.names(resTableDOWNc) = row.names(diffRes)

colnames(resTableUP)   = paste0("select_", colnames(diffRes)[seq(1,ncol(diffRes), by = 2)])
colnames(resTableDOWN)   = paste0("select_", colnames(diffRes)[seq(1,ncol(diffRes), by = 2)])

colnames(resTableUPc)  = paste0("select_", colnames(diffResC)[seq(1,ncol(diffResC), by = 2)])
colnames(resTableDOWNc)  = paste0("select_", colnames(diffResC)[seq(1,ncol(diffResC), by = 2)])

# Apply filter to select genes
k = 1
for(i in seq(1,ncol(diffRes), by = 2)){
  resTableUP[,k]   = (diffRes[,i] > T1) & (diffRes[,(i+1)] < T2)
  resTableDOWN[,k] = (diffRes[,i] < -T1) & (diffRes[,(i+1)] < T2)

  resTableUPc[,k] = (diffResC[,i] > T1) & (diffResC[,(i+1)] < T2)
  resTableDOWNc[,k] = (diffResC[,i] < -T1) & (diffResC[,(i+1)] < T2)

  k = k + 1
  # end of for()
}

# Number of times a gene is differentially expressed
# in all comparisons.
TotalDiff  = apply(cbind(resTableUP, resTableDOWN), 1, sum)
TotalDiffC = apply(cbind(resTableUPc, resTableDOWNc), 1, sum)

# Is the gene DE for the three time points in the intermediate time points ?
MiddleDiffUP    = apply(resTableUP[,2:4], 1, sum)
MiddleDiffDOWN  = apply(resTableDOWN[,2:4], 1, sum)

# Same for control...
MiddleDiffUPc    = apply(resTableUPc[,2:4], 1, sum)
MiddleDiffDOWNc  = apply(resTableDOWNc[,2:4], 1, sum)

# Find genes with identical deregulation in both experiments
filterSupp = rep(NA, nrow(diffRes))
for(i in 1:nrow(diffRes)){
  if((sum(resTableUP[i,] == resTableUPc[i,]) == 5) & (sum(resTableDOWN[i,] == resTableDOWNc[i,]) == 5)){
    filterSupp[i] = "YES"
  }
}

# Combine all results
expData3 = cbind(numPeaks, whichPeaks, coordPeaks,
                 TotalDiffC, MiddleDiffUPc, TotalDiff, MiddleDiffUP, filterSupp,
```

```
                 expData, expData2)

write.table(expData3, file = "data_integration.txt", sep = "\t", quote = F)

#####
# Part 4 : Search for RDRM in promoters of genes
#####

fimoData = read.csv("FIMO_1.txt", header = T, sep = "\t")

numMotif    = NULL
whichMotif  = NULL

# To store motifs positions
coordMotif  = NULL

for(i in 1:nrow(expData2)){
  if(sum(fimoData[,3] == row.names(expData2)[i]) == 0){
    numMotif   = c(numMotif, 0)
    whichMotif = c(whichMotif, NA)
    coordMotif = c(coordMotif, NA)
  }else if(sum(fimoData[,3] == row.names(expData2)[i]) == 1){
    numMotif   = c(numMotif, 1)
    whichMotif = c(whichMotif, fimoData[fimoData[,3] == row.names(expData2)[i],9])
    # To store peak location
    coordMotif = c(coordMotif, paste(fimoData[fimoData[,3] == row.names(expData2)[i],3:6], collapse = "|"))
  }else if(sum(fimoData[,3] == row.names(expData2)[i]) > 1){
    numMotif   = c(numMotif, sum(fimoData[,3] == row.names(expData2)[i]))
    whichMotif = c(whichMotif, paste(fimoData[fimoData[,3] == row.names(expData2)[i],9], collapse = "|"))
    coordMotif = c(coordMotif, paste(fimoData[fimoData[,3] == row.names(expData2)[i],3:6], collapse = "|"))
  }

  # end of for()
}

# to store the results
numMotif1    = numMotif
whichMotif1  = whichMotif
coordMotif1  = coordMotif

fimoData = read.csv("FIMO_2.txt", header = T, sep = "\t")

numMotif    = NULL
whichMotif  = NULL

# To store motifs positions
coordMotif  = NULL

for(i in 1:nrow(expData2)){
  if(sum(fimoData[,3] == row.names(expData2)[i]) == 0){
    numMotif   = c(numMotif, 0)
    whichMotif = c(whichMotif, NA)
    coordMotif = c(coordMotif, NA)
  }else if(sum(fimoData[,3] == row.names(expData2)[i]) == 1){
    numMotif   = c(numMotif, 1)
    whichMotif = c(whichMotif, fimoData[fimoData[,3] == row.names(expData2)[i],9])
    # To store peak location
    coordMotif = c(coordMotif, paste(fimoData[fimoData[,3] == row.names(expData2)[i],3:6], collapse = "|"))
  }else if(sum(fimoData[,3] == row.names(expData2)[i]) > 1){
    numMotif   = c(numMotif, sum(fimoData[,3] == row.names(expData2)[i]))
    whichMotif = c(whichMotif, paste(fimoData[fimoData[,3] == row.names(expData2)[i],9], collapse = "|"))
    coordMotif = c(coordMotif, paste(fimoData[fimoData[,3] == row.names(expData2)[i],3:6], collapse = "|"))
  }

  # end of for()
}

# to store the results
numMotif2    = numMotif
whichMotif2  = whichMotif
coordMotif2  = coordMotif

fimoData = read.csv("FIMO_3.txt", header = T, sep = "\t")

numMotif    = NULL
whichMotif  = NULL
```

```
# To store motifs positions
coordMotif  = NULL

for(i in 1:nrow(expData2)){
  if(sum(fimoData[,3] == row.names(expData2)[i]) == 0){
    numMotif   = c(numMotif, 0)
    whichMotif = c(whichMotif, NA)
    coordMotif = c(coordMotif, NA)
  }else if(sum(fimoData[,3] == row.names(expData2)[i]) == 1){
    numMotif   = c(numMotif, 1)
    whichMotif = c(whichMotif, fimoData[fimoData[,3] == row.names(expData2)[i],9])
    # To store peak location
    coordMotif = c(coordMotif, paste(fimoData[fimoData[,3] == row.names(expData2)[i],3:6], collapse = "|"))
  }else if(sum(fimoData[,3] == row.names(expData2)[i]) > 1){
    numMotif   = c(numMotif, sum(fimoData[,3] == row.names(expData2)[i]))
    whichMotif = c(whichMotif, paste(fimoData[fimoData[,3] == row.names(expData2)[i],9], collapse = "|"))
    coordMotif = c(coordMotif, paste(fimoData[fimoData[,3] == row.names(expData2)[i],3:6], collapse = "|"))
  }

  # end of for()
}

# to store the results
numMotif3    = numMotif
whichMotif3  = whichMotif
coordMotif3  = coordMotif

fimoData = read.csv("FIMO_4.txt", header = T, sep = "\t")

numMotif    = NULL
whichMotif  = NULL

# To store motifs positions
coordMotif  = NULL

for(i in 1:nrow(expData2)){
  if(sum(fimoData[,3] == row.names(expData2)[i]) == 0){
    numMotif   = c(numMotif, 0)
    whichMotif = c(whichMotif, NA)
    coordMotif = c(coordMotif, NA)
  }else if(sum(fimoData[,3] == row.names(expData2)[i]) == 1){
    numMotif   = c(numMotif, 1)
    whichMotif = c(whichMotif, fimoData[fimoData[,3] == row.names(expData2)[i],9])
    # To store peak location
    coordMotif = c(coordMotif, paste(fimoData[fimoData[,3] == row.names(expData2)[i],3:6], collapse = "|"))
  }else if(sum(fimoData[,3] == row.names(expData2)[i]) > 1){
    numMotif   = c(numMotif, sum(fimoData[,3] == row.names(expData2)[i]))
    whichMotif = c(whichMotif, paste(fimoData[fimoData[,3] == row.names(expData2)[i],9], collapse = "|"))
    coordMotif = c(coordMotif, paste(fimoData[fimoData[,3] == row.names(expData2)[i],3:6], collapse = "|"))
  }

  # end of for()
}

# to store the results
numMotif4    = numMotif
whichMotif4  = whichMotif
coordMotif4  = coordMotif

expData4 = cbind(expData3, numMotif1, whichMotif1, coordMotif1, numMotif2, whichMotif2, coordMotif2,
                 numMotif3, whichMotif3, coordMotif3, numMotif4, whichMotif4, coordMotif4)
write.table(expData4, file = "data_integration_V4.txt", sep = "\t", quote = F)
# That's all
```
