# Supplementary material for: Characterization of the Radiation Desiccation Response Regulon of the Radioresistant Bacterium Deinococcus radiodurans by Integrative Genomic Analyses
Source: Cells. 2021 Sep 25;10(10):2536. doi: 10.3390/cells10102536 (PMC8533742; doi:10.3390/cells10102536)
Supplement: Supplementary file 1 [file cells-10-02536-s001.zip › cells-1325232-supplementary publish check/upload/Table S1.pdf]

Table S1. Bacterial strains and plasmids used in this study

Strains	Description	Source or reference
E. coli		
DH5α	*supE44ΔlacU(Φ 80lacZΔM15) hsdR17 recA1 endA1 gyrA96 thi-1 relA1*	Laboratory stock
SCS110	*endA dam dcm supE44 Δ (lac-proAB) (F'traD36proAB lacIq ZΔM15)*	Laboratory stock
D. radiodurans		
R1/ GY 9613	ATCC 13939	Laboratory stock
GY 14125	Non homogenotized *ΔddrOΩcat*	[20]
GY 15910	*hflX::HAΩkan*	[76]
GY 15950	*ΔirrEΩcat*	[21]
GY 16977	*dr0001::v5Ωkan*	This work
GY 16978	*ΔirrEΩcat dr0001::v5Ωkan*	This work
GY 16979	*ddrN::v5Ωkan*	This work
GY 16980	*ΔirrEΩcat ddrN::v5Ωkan*	This work
GY 16925	*recA::HAΩkan ΔddrOΩcat* / p11891 ($ddrO^{+}$/$repU_{TS}$)	This work
GY 17357	*ΔddrOΩcat* / p17237	This work
GY 17370	*ΔddrOΩcat* / p17238	This work
GY 17375	*ddrD::HAΩkan ΔddrOΩcat* / p17238 ($ddrO^{+}$/repU_{TS})	This work
GY 17381	*pprA::HAΩkan ΔddrOΩcat* / p17238 ($ddrO^{+}$/repU_{TS})	This work
GY 17393	*ΔddrOΩcat* / p17245 (*ddrO*-FLAG/$repU_{TS}$)	This work
GY 17960	*ΔirrEΩcat dr0216::v5Ωkan*	This work
GY 17962	*ΔirrEΩcat dr0217::v5Ωkan*	This work
GY 17964	*ΔirrEΩcat dr2255::v5Ωkan*	This work
GY 17966	*ΔirrEΩcat drC0017::v5Ωkan*	This work
GY 17968	*ΔirrEΩcat drC0023::v5Ωkan*	This work
GY 17988	*drC0017::v5Ωkan*	This work
GY 17994	*dr0217::v5Ωkan*	This work
GY 17996	*dr2255::v5Ωkan*	This work
GY 18218	*ddrO::v5Ωkan*	This work
GY 18303	*drA0166::v5Ωkan*	This work
GY 18305	*dr2173::v5Ωkan*	This work
GY 18307	*dr0614::v5Ωkan*	This work
GY 18310	*dr1297::v5Ωkan*	This work
GY 18311	*dr2410::v5Ωkan*	This work
GY 18313	*dr0561::v5Ωkan*	This work

GY 18317	*ddrF::v5Ωkan*	This work
GY 18323	*ΔirrEΩcat drA0166::v5Ωkan*	This work
GY 18325	*ΔirrEΩcat dr2173::v5Ωkan*	This work
GY 18327	*ΔirrEΩcat dr0614::v5Ωkan*	This work
GY 18330	*ΔirrEΩcat dr1297::v5Ωkan*	This work
GY 18331	*ΔirrEΩcat dr2410::v5Ωkan*	This work
GY 18333	*ΔirrEΩcat dr0561::v5Ωkan*	This work
GY 18337	*ΔirrEΩcat ddrF::v5Ωkan*	This work
GY 18341	*recG::v5Ωkan*	This work
GY 18343	*ligA(dr2069)::v5Ωkan*	This work
GY 18345	*ΔirrEΩcat recG::v5Ωkan*	This work
GY 18347	*ΔirrEΩcat ligA::v5Ωkan*	This work
GY 18364	*ddrR::v5Ωkan*	This work
GY 18366	*ΔirrEΩcat ddrR::v5Ωkan*	This work
GY 18368	*mutL::v5Ωkan*	This work
GY 18370	*ΔirrEΩcat mutL::v5Ωkan*	This work
GY 18372	*irrI::v5Ωkan*	This work
GY 18374	*ΔirrEΩcat irrI::v5Ωkan*	This work
GY 18376	*dr1571::v5Ωkan*	This work
GY 18378	*ΔirrEΩcat dr1571::v5Ωkan*	This work
GY 18380	*dr1572::v5Ωkan*	This work
GY 18382	*ΔirrEΩcat dr1572::v5Ωkan*	This work
GY 18384	*frnE(dr0659)::v5Ωkan*	This work
GY 18386	*ΔirrEΩcat frnE::v5Ωkan*	This work
GY 18388	*rsr(dr1262)::v5Ωkan*	This work
GY 18392	*dr1482::v5Ωkan*	This work
GY 18394	*ΔirrEΩcat dr1482::v5Ωkan*	This work
GY 18505	*dr0216::v5Ωkan*	This work
GY 18511	*hutU::v5Ωkan*	This work
GY 18514	*ΔirrEΩcat hutU::v5Ωkan*	This work
GY 18545	*dr0685::v5Ωkan*	This work
GY 18547	*ΔirrEΩcat dr0685::v5Ωkan*	This work
GY 18549	*dr217-::v5Ωkan*	This work
GY 18551	*ΔirrEΩcat dr2174::v5Ωkan*	This work
GY 18557	*ΔirrEΩcat hflX::HAΩkan*	This work

**Plasmids**	**Description**	**Source or reference**
pGTC101	Source of chloramphenicol cassette in *D. radiodurans*	Laboratory stock
p11806	Source of kanamycin cassette in *D. radiodurans*	Laboratory stock
p11554	Shuttle vector *E. coli* / *D. radiodurans*, $Spec^R$	Laboratory stock
p12625	Source of hygromycin cassette in *D. radiodurans*	Laboratory stock
p12723	Source of FLAG cassette in *D. radiodurans*	Laboratory stock
p12764	Source of HA cassette in *D. radiodurans*	Laboratory stock

p11830	Vector thermosensitive for replication in *D. radiodurans* Spec^{R}, p*repU*$_{Ts}$	[53]
p11891	p13841: p*repU*$_{TS}$::*ddrO*	[20]
p13841	Vector thermosensitive for replication in *D. radiodurans* Spec^{R}, p*repU*$_{Ts}$, P_{Spac}-term116	[53]
p17235	p11554 + multiple site of clonage of p13841	This work
p17236	p11830 + multiple site of clonage of p13841	This work
p17237	p17335 : p*repU*::*ddrO*	This work
p17238	p17336 : p*repU*$_{TS}$::*ddrO*	This work
p17245	p17236 : p*repU*$_{TS}$::*ddrO*-FLAG	This work
