# Supplementary material for: Characterization of the Radiation Desiccation Response Regulon of the Radioresistant Bacterium Deinococcus radiodurans by Integrative Genomic Analyses
Source: Cells. 2021 Sep 25;10(10):2536. doi: 10.3390/cells10102536 (PMC8533742; doi:10.3390/cells10102536)
Supplement: Supplementary file 1 [file cells-10-02536-s001.zip › cells-1325232-supplementary publish check/upload/Table S2.pdf]

Table S2: Primers used for strains and plasmids constructions. Restriction sites are indicated in bold.

Primers	Primer sequence	Amplified fragment
**Primers for cloning *ddrO* for complementation**		
NE26	TAC**GGATCC**GCGCTCACAATTAAGCTTG	Multicloning sites from p13841
NE27	TCAC**AGATCT**GCCGTTCGCGAACTCGAGTG	
NE28	TCA**GGATCC**AGCGGCCAATTTCTGGTTTC	*D. radiodurans ddrO* and its promoter
NE29	AT**GCGGCCGC**GGAGTTGTGCGGAAATCAG	
HH134	GGAC**TTTAAA**TTACTACTTGTCATCGTCATCCTTG	*ddrO*-FLAG and its promoter
HH202	GCCTGAGTCCATATGACATTGAAACTGCACGAAC	
**Primers for construction of *ddrO-v5 Ωkan* fragment**		
PS611	GTGCCTGATTCTGCCCACAAG	*ddrO* 3' region with 5' region of the v5 sequence (complementary sequence with PS465-NE212 fragment)
NE211	GGCCCAGCAGCGGGTTCGGGATCGGCTTGCCGTTCAGGATGCGTTTGAGATG	
PS465	GCTCTTCGTGGCAGGCACAG	Downstream region of *ddrO* with 3' region of the v5 sequence (complementary sequence with PS611-NE211 fragment)
NE212	GAACCCGCTGCTGGGCCTGGACAGCACCTGATTTCCGCACAACTC	
ddrO-v5 fragment is produced and amplified by fusion PCR (PS611-NE211 fragment + PS465-NE212 fragment)		
Kan5	GGAA**GGATCC**GCATTCTGCCTCCAGCATCTC	*kan* fragment
Kan3	GGAA**TCTAGA**GCAAGCAGCAGATTACG	
PS 465	GCTCTTCGTGGCAGGCACAG	Downstream region of *ddrO*
NE14	TAAT**TCTAGA**CTGTGAGGAGGCGTGTCAGG	
**Primers for construction of v5-tagged protein**		
NE281	TCAT**TCTAGA**GGCAAGCCGATCCCGAACCC	*v5 Ωkan* fragment
NE238	TCAT**CTCGAG**GGTCTGACGCTCAGTGGAAC	
NE249	CCCGGCGTAGACGGCCCCAA	Upstream region of *drA0166*
NE250	TCAT**CTCGAG**TCTGCGCCTGCTGAAGCTGA	
NE284	GGGCCTCAGTGTCTGGATTG	Fragment with 3' *drA0166* region
NE285	TCAT**TCTAGA**GCGCAACTGTTCCAGAATCG	
NE254	TCAT**CTCGAG**TCTCCCGGGTGGCCTACAAC	Upstream region of *dr2173*
NE255	AGCTGCACGCCCACTTCTTC	
NE286	ACCTCTACGGTTGCGATTTC	Fragment with 3' *dr2173* region
NE287	TCAT**TCTAGA**GGTACAGTCCCAGTTGTAGG	
NE257	GGCCCACAATCAGGTCCATC	

NE258	TCAT**CTCGAG**CATGACCCAGCCCTCCATCC	Upstream region of *dr0614*
NE288	GCTTCGTGCAAGACCAGGAC	Fragment with 3’ *dr0614* region
NE289	TCAT**TCTAGA**GTCTGCCCCGTCGCCGAAG	
NE261	CGACGCCTTCTTCCTGGTTG	Upstream region of *dr1297*
NE262	TCAT**CTCGAG**GAGCTCTGGGCAAATTCAGG	
NE290	GCGCCATTCGCACCGATTTC	Fragment with 3’ *dr1297* region
NE291	TCAT**TCTAGA**GTGCTCCTTACGGGGCAGCAG	
NE265	ACCGGCTTTTCGGGCAGTG	Upstream region of *dr2410*
NE266	TCAT**CTCGAG**GAAGACACCGCCGAAGCCTG	
NE369	AACTGTACCTCGGCGAAGTG	Fragment with 3’ *dr2410* region
NE293	TCAT**TCTAGA**GGCTTCGGCGGTGTCTTCG	
NE269	CGGTAAAGGCGAGCACGAAC	Upstream region of *dr0561*
NE270	TCAT**CTCGAG**GCCGTGGGTGAGATTAAGGG	
NE294	CGGTCAAAGCGGCGAGTTTC	Fragment with 3’ *dr0561* region
NE295	TCAT**TCTAGA**CTTGATATTGCCCTTAATCTCAC	
NE273	CGTTCGACTACGGCCTCTAC	Upstream region of *ddrF*
NE274	TCAT**CTCGAG**CTTCCAGCGCCAACTTATCC	
NE296	TTACGTACTCACGCGATCAG	Fragment with 3’ *ddrF* region
NE297	TCAT**TCTAGA**ACTGAGGTGCAAGTTTGACAAC	
SI276	GCGCAACAGTTCAGTGTAAG	Upstream region of *recG*
NE303	TCAT**CTCGAG**TTTGCCGCTAGGACCGCTGG	
NE304	CGCTGCCGAGAAGGATTACG	Fragment with 3’ *recG* region
NE305	TCAT**TCTAGA**AATCACTTCGCGGTACGCCACG	
NE306	CAGCTTGAGGCGAGCAAGAC	Upstream region of *ligA*
NE307	TCAT**TCTAGA**GCTTTCAGCGGGGGCCGCCTCAG	
NE308	GAGGGTTTGACGCTGCTCTG	Fragment with 3’ *ligA* region
NE309	TCAT**CTCGAG**TGCTACCCTAGTCTGACAAG	
NE323	AGCAGCACGTGGTTGTC	Upstream region of *dr0216*
NE324	TCAT**CTCGAG**GATTCTGATGCGCCTCAAGC	
NE325	ATTGGCGGCATGACTGCTCC	Fragment with 3’ *dr0216* region
NE326	TCAT**TCTAGAG**CCCTCTGCGCCCGCCACGTC	
PS865	GCG**TCTAGA**TTCCTCGCTGTAGGTCTTTTCG	Upstream region of *dr0217*
PS866	GAAGGCCGCGAAATGACCAC	
NE329	ACAGCGGTTTCTTCGTCCAG	Fragment with 3’ *dr0217* region
NE330	TCAT**CTCGAG**GCTCGCCTGAGCGATGAAAC	
NE406	CACTTTCGGTGGTGGGTTCC	Upstream region of *dr2255*
NE407	TCAT**TCTAGA**CGCCTCGCGCTCCCAGGCCAC	
NE408	TTGCCAGGCGTTGCGAAAGC	Fragment with 3’ *dr2255* region
NE409	TCAT**CTCGAG**TCGTCGTTCACCCGGAGCAC	
NE340	CGAGGCCCTGTTTCTTGAGC	Upstream region of *drC0017*
NE341	TCAT**CTCGAG**GGTGGACGCATGACACAGAC	

NE342	TCATCCGCCAGCAGTACGAG	Fragment with 3' *drC0017* region
NE343	TCAT**TCTAGA**TGCGTCCACCTCGACCTCCAG	
NE346	ACGGCGAAAGCTCAGTTGCG	Upstream region of *drC0021*
NE347	TCAT**CTCGAG**TCAGTCCCTCACTCCAGTTC	
NE348	TTGGAACCGGGAGTGGGAAC	Fragment with 3' *drC0021* region
NE349	TCAT**TCTAGA**CTCCTCTCTGTTCTGCCGGAGCC	
NE350	AGGCCGTCATAGAGCGTCTG	Upstream region of *dr1262 (rsR)*
NE351	TCAT**TCTAGA**AACCTCGCCCCGCGCAAAAG	
NE352	TGCCGTGACTGTCGGTGATG	Fragment with 3' *dr1262 (rsR)* region
NE353	TCAT**CTCGAG**GCCACGCACCATCTCTAACG	
NE354	GCCTGGCGATGAAGTACAGC	Upstream region of *dr0659 (frnE)*
NE355	TCAT**TCTAGA**GTTGTTAGGGCGCTGGGGCACC	
NE356	TCAAAGTGCCCGTGCGTCAG	Fragment with 3' *dr0659(frnE)* region
NE357	TCAT**CTCGAG**CGAGGCTGCTCTGGCGAAAG	
NE358	CCGACCAGATCGCCTTTCAG	Upstream region of *drA0151 (hutU)*
NE359	TCAT**TCTAGA**CGCGTGATCCTTGATGCCCAG	
NE360	TTGTCGTAGGCGCTCTGACC	Fragment with 3' *drA0151(hutU)* region
NE368	TCA**TCTCGAG**GTCTGGGCATCAAGGATCAC	
NE364	GAAACTCGGCCTTGATTTCC	Upstream region of *dr1482*
NE365	TCAT**CTCGAG**GACGCCCTGAAGGGTAATGG	
NE366	TCGTCATGGGCAAGCACTCG	Fragment with 3' *dr1482* region
NE367	TCAT**TCTAGA**GGGCGTCATCTGCGTGATCG	
NE388	CACCATCAAGCCCGGTGAAG	Upstream region of *dr0685*
NE389	GAGG**TCTAGA**GTTCACCTTGACCTGGTAG GTCACGGTGATGG	
NE390	CTCGTTGACCGCTTCGAGTG	Fragment with 3' *dr0685* region
NE391	TCAT**CTCGAG**CCTACCAGGTCAAGGTGAAC	
NE394	ATCAACGGCCCGAGTAAGTG	Upstream region of *dr2174*
NE395	TCGG**TCTAGA**CTTCGCCACGATGTTGATAATC	
NE396	CGTTATTTCTTGGGCTTGGG	Fragment with 3' *dr2174* region
NE397	TCAT**CTCGAG**GACTTAATCCGAGCGGAGTG	
EB154	AAAT**TCTAGA**CCCCAGCGGCGGCAGGTTGC	Upstream region of *ddrR*
EB155	TACGCTGAGTCCATCAGGAG	
EB156	GGAA**CTCGAG**GCGTGCCTTCTTTGCAAGTC	Fragment with 3' *ddrR* region
EB157	CCGTGTCGGGGTCGCCAGAG	
EB158	AAAT**TCTAGA**AACCACGCTGTCACGCCCCC	Upstream region of *mutL*
EB159	CGTCTATCAGGAGCTGTACC	
EB160	GGAA**CTCGAG**ATGTGGGTGGCTTGAGTGCG	Fragment with 3' *mutL* region
EB161	TCACAGCCGGTCAGTGTAAG	
EB162	AAAT**TCTAGA**GTGAGCTTTCTCTGTGCTGG	Upstream region of *irrI*
EB163	CCTCAACACCGTGAAGTATC	
EB164	GGAA**CTCGAG**CCTCCACGCCTGCCAGGTC	

EB165	TGTACGCCGAAATCGAGAAG	Fragment with 3' *irrI* region
CB138	TGGTGGATAAGGCGGGCAAC	Upstream region of *dr1571*
CB139	ACGT**TCTAGA**GCGCTTGATCCAGGTCAGCGCGATG	
CB140	TCAT**CTCGAG**GAGGTCCTGAGGACGCCTAG	Fragment with 3' *dr1571* region
CB141	ATCGACACGGCGAAGTGCAG	
CB142	TATTCGCCGCTGCTCTACGC	Upstream region of *dr1572*
CB143	ACGT**TCTAGA**GCGCACCAGCGGGTGCAGCTCC	
CB144	TCAT**CTCGAG**CGCTGAGGGGTGGCTCACTG	Fragment with 3' *dr1572* region
CB145	GAATGTCGAGGTCGAGAAAG	
ddrN11	AT**TCTAGA**GCAGCGCCCTGTGAGGAGCG	Upstream region of *ddrN*
ddrN5	AGCTTGAAGCGCCGTCTGAG	
ddrN12	AAT**CTCGAG**GCTGACGGGACGCTGCTGAC	Fragment with 3' *ddrN* region
ddrN4	ACATTGAGCGCCTCACCGTC	
CB146	TGGCTATCCGCGACTTTCCG	Upstream region of *dr0001*
CB147	ACGT**TCTAGA**AACGCGCAGCGTGACCATGACC	
CB148	TCAT**CTCGAG**TAAGGGGCCTTCTGAGGCCGTT	Fragment with 3' *dr0001* region
CB149	TAGCGGTAGAGCGGGATGTC	
