# Supplementary material for: Characterization of the Radiation Desiccation Response Regulon of the Radioresistant Bacterium Deinococcus radiodurans by Integrative Genomic Analyses
Source: Cells. 2021 Sep 25;10(10):2536. doi: 10.3390/cells10102536 (PMC8533742; doi:10.3390/cells10102536)
Supplement: Supplementary file 1 [file cells-10-02536-s001.zip › cells-1325232-supplementary publish check/upload/Table S6.pdf]

Table S6. Number of unique and common up- and downregulated genes in W37 and D37, respectively during the time course.

For each time lapse, a 2-fold change expression threshold for the ratio experiments was applied together with a *p-Value* <0.01.

	1 h-4 h	1 h-6 h	1 h-8 h	1 h-16 h	1 h-24 h
**Total in W37**	350	679	993	1303	1501
**Up-regulated in W37**	204	332	474	620	732
**Down-regulated in W37**	146	347	519	683	769
**Total in D37**	358	1011	1177	1539	1536
**Up-regulated in D37**	253	576	650	818	788
**Down-regulated in D37**	105	435	527	721	748
**Common genes in W37 and D37**	159	501	660	912	1125
**Up-regulated in W37**	106	276	339	455	569
**Down-regulated in W37**	53	225	321	457	556
**Up-regulated in D37**	109	290	368	436	573
**Down-regulated in D37**	50	211	292	476	552
